# Bipolar Androgen Therapy as a Potential Mechanistic Bridge to Enhance PARP Inhibitor Efficacy in Prostate Cancer

**DOI:** 10.1002/kjm2.70292

**Published:** 2026-09-17

**Authors:** Jhen‐Hao Jhan, Jiun‐Hung Geng, Hung‐Lung Ke, Shu‐Pin Huang, Li Jia

**Affiliations:** ^1^ Department of Urology Brigham and Women's Hospital & Harvard Medical School Boston Massachusetts USA; ^2^ Department of Urology Kaohsiung Municipal Siaogang Hospital Kaohsiung Taiwan; ^3^ Department of Urology Kaohsiung Medical University Hospital Kaohsiung Taiwan; ^4^ Graduate Institute of Clinical Medicine, College of Medicine Kaohsiung Medical University Kaohsiung Taiwan; ^5^ Department of Urology, School of Medicine, College of Medicine Kaohsiung Medical University Kaohsiung Taiwan; ^6^ Department of Urology Kaohsiung Medical University Gangshan Hospital Kaohsiung Taiwan; ^7^ School of Post‐Baccalaureate Medicine, College of Medicine Kaohsiung Medical University Kaohsiung Taiwan; ^8^ Institute of Medical Science and Technology, College of Medicine National Sun Yat‐Sen University Kaohsiung Taiwan

**Keywords:** bipolar androgen therapy (BAT), homologous recombination repair (HRR), PARP inhibitor, prostate cancer, supraphysiological androgen (SPA)

## Abstract

Prostate cancer remains a leading cause of cancer‐related mortality, largely driven by progression to metastatic castration‐resistant prostate cancer (mCRPC). Although poly(ADP‐ribose) polymerase inhibitors (PARPis) have improved outcomes in patients with homologous recombination repair (HRR) alterations, particularly in *BRCA2*‐mutated disease, their clinical benefit is limited by restricted patient selection, modest efficacy in non‐*BRCA* HRR alterations, and the frequent emergence of resistance. These limitations highlight an unmet need for strategies that can both expand the therapeutic population and overcome PARPi resistance. Bipolar androgen therapy (BAT), which alternates between supraphysiological and near‐castrate androgen exposure, has emerged as a paradoxical yet clinically active approach in mCRPC. Unlike conventional androgen deprivation strategies, preclinical evidence suggests that BAT induces acute androgen receptor–mediated DNA damage while simultaneously suppressing HRR gene expression. This dual effect may generate a transcription‐coupled homologous recombination–deficient state that is independent of canonical baseline genomic HRR alterations, thereby potentially sensitizing tumors to PARP inhibition. Current clinical trials of BAT combined with PARP inhibitors suggest activity in both HRR‐deficient and HRR‐proficient disease. Collectively, these findings suggest a preliminary, hypothesis‐generating conceptual framework in which BAT may expand the therapeutic scope of PARPis beyond genomically defined HRR‐mutated tumors and may help counteract mechanisms of PARPi resistance in mCRPC.

Prostate cancer ranks among the most common causes of cancer‐related mortality in men worldwide [1], with most deaths occurring after progression to metastatic castration‐resistant prostate cancer (mCRPC). Patients with mCRPC harboring homologous recombination repair (HRR) mutations, particularly *BRCA2* alterations, benefit from poly(ADP‐ribose) polymerase inhibitors (PARPis), which have demonstrated efficacy both as monotherapy after progression on androgen receptor pathway inhibitors (ARPIs) and in first‐line combination with ARPIs. Accordingly, the US Food and Drug Administration approved olaparib and rucaparib monotherapy in 2020 for biomarker‐selected mCRPC in the second‐line setting, followed in 2023 by three PARPi–ARPI combinations—olaparib plus abiraterone, niraparib plus abiraterone, and talazoparib plus enzalutamide—for first‐line treatment. Table 1 summarizes the current FDA‐approved indications and key supporting trials.

**TABLE 1:** FDA‐approved PARP inhibitors for mCRPC.

Drug	Setting	Indications	Year of approval	Related trials	Biomarkers
					(germline or somatic mutations)
Olaparib	2nd line	mCRPC patients with deleterious HRR gene mutation (germline and/or somatic), who have progressed following prior treatment with enzalutamide or abiraterone.	2020	PROfound (NCT02987543)	*ATM, BRCA1, BRCA2, BARD1, BRIP1, CDK12, CHEK1, CHEK2, FANCL, PALB2, RAD51B, RAD51C, RAD51D, RAD54L*
Rucaparib	2nd line	mCRPC patients with deleterious BRCA mutation (germline and/or somatic), who have been treated with androgen receptor‐directed therapy and a taxane‐based chemotherapy.	2020	TRITON2 (NCT02952534)	*BRCA1, BRCA2*
Olaparib + Abiraterone	1st line	mCRPC patients with deleterious BRCA mutation, as determined by an FDA‐approved companion diagnostic test.	2023	PROpel (NCT03732820)	*BRCA1, BRCA2*
Niraparib + Abiraterone	1st line	mCRPC patients with deleterious BRCA mutation, as determined by an FDA‐approved companion diagnostic test.	2023	MAGNITUDE (NCT03748641) Cohort 1	*BRCA1, BRCA2*
Talazoparib + Enzalutamide	1st line	mCRPC patients with HRR gene mutation	2023	TALAPRO‐2 (NCT03395197)	*BRCA1*, *BRCA2*, *PALB2*, *ATM*, *ATR*, *CHEK2*, *FANCA*, *RAD51C*, *NBN*, *MLH1*, *MRE11A*, *CDK12*

The antitumor activity of PARPis is primarily mediated by synthetic lethality. By inhibiting PARP1‐dependent single‐strand break repair, these agents promote double‐strand break (DSB) formation through two non–mutually exclusive mechanisms: PARP1 trapping and a “gap” model involving post‐replicative single‐stranded DNA gaps [2]. In HRR‐intact tumors, DSBs are efficiently repaired via BRCA1/2‐dependent, RAD51‐mediated homologous recombination and ATR–CHK1 and ATM–CHK2 signaling. In contrast, HRR‐deficient tumors fail to resolve these lesions, leading to genomic instability and cell death.

Despite the demonstrated survival benefit of PARPis in HRR‐altered mCRPC, their clinical utility remains limited. First, robust efficacy is largely confined to *BRCA2*‐mutated tumors, whereas benefit in other HRR alterations (e.g., *CDK12*, *ATM*, *CHEK2*) is inconsistent, suggesting these alterations may not confer sufficient homologous recombination deficiency (HRD) for synthetic lethality—raising concerns that single‐gene biomarker strategies may not adequately reflect functional HRD beyond *BRCA*. Second, HRR alterations are relatively uncommon; although *BRCA2* is the most frequently altered HRR gene in mCRPC, it accounts for only about 10% of prostate cancer cases [3]. Third, even among *BRCA2*‐mutated tumors, resistance is common, with responses in only approximately 50% of patients, including those with homozygous deletions [4, 5]. These limitations underscore the need for refined HRD definitions, improved biomarker strategies, and approaches to overcome resistance.

Substantial efforts have focused on expanding PARPi‐eligible populations and overcoming resistance. Our previous work identified *TP53* and *RB1* as key modulators of PARPi response: disruption of the *CHEK2–TP53* axis restores *BRCA2* expression, whereas *RB1* loss—often co‐occurring with *RNASEH2B* or *BRCA2* deletions—induces resistance via E2F1‐mediated *BRCA2* upregulation; in both contexts, ATR inhibition suppresses *BRCA2* and re‐sensitizes tumors to PARPis [6]. Additional preclinical strategies further support pharmacologic re‐sensitization. Targeting the CDT1–geminin axis restores PARPi sensitivity in *BRCA2*‐independent resistance models [5]; EZH2‐mediated PARP1 methylation supports combined EZH2–PARP1 inhibition [7]; and CDK12/7/9 inhibition can induce a functional “BRCAness” state in HRR‐intact tumors [8]. Although several of these strategies have entered early‐phase clinical investigation, robust clinical benefit in unselected populations has not yet been established, underscoring the persistent gap between mechanistic rationale and clinical validation.

Physiological levels of androgens promote prostate cancer growth through androgen receptor (AR) signaling. Paradoxically, supraphysiological androgen (SPA) suppresses tumor growth, particularly after prolonged androgen deprivation therapy (ADT). Based on this observation, two pioneering Phase I trials first introduced SPA into clinical practice and demonstrated its safety in patients with mCRPC [9, 10]. This approach evolved into bipolar androgen therapy (BAT), which alternates serum testosterone between supraphysiological (> 1000 ng/dL) and near‐castrate (~100 ng/dL) levels in 28‐day cycles.

Unlike conventional strategies that maintain castrate testosterone levels using ADT or ARPIs, BAT induces rapid hormonal oscillations that disrupt adaptive AR upregulation, a key mechanism of resistance in CRPC. This fluctuation induces cellular stress and DNA damage while exerting broader biological effects. A pilot clinical study demonstrated PSA declines in approximately 50% of CRPC patients and resensitization to subsequent ADT [11]. These findings were confirmed in Phase II trials, including RESTORE and TRANSFORMER, which demonstrated clinical activity and favorable safety profiles [12, 13]. The Phase II COMBAT trial further showed that BAT induces pro‐inflammatory gene expression and enhances antitumor immunity when combined with immune checkpoint inhibition [14]. In the context of limitations of PARPi therapy, BAT represents a clinically promising and mechanistically distinct strategy [15]; it should be noted, however, that this reflects a difference in the stage of clinical development rather than superiority of mechanistic evidence or proven efficacy, as BAT itself has not yet been evaluated in randomized Phase III trials.

Early preclinical studies demonstrated that SPA induces AR‐dependent replication stress and DSBs, partly through TOP2B‐mediated DNA cleavage and disruption of DNA replication licensing, thereby promoting genomic instability and TMPRSS2–ERG rearrangements [16, 17]. Emerging evidence suggests that transcriptional hyperactivation by AR provides an additional source of replication stress. Excessive AR‐driven transcription can promote R‐loop accumulation, particularly at highly transcribed genes and sites of RNA polymerase II pausing [18]. R‐loops are three‐stranded nucleic acid structures in which nascent RNA hybridizes with the template DNA strand, forming an RNA–DNA hybrid and leaving the non‐template DNA strand single‐stranded. Although R‐loops have physiological functions in transcriptional regulation, their persistent accumulation can impede replication‐fork progression and generate transcription–replication conflicts, leading to fork stalling, fork collapse, and DSB formation. These conflicts have also been implicated in tumor‐cell sensitivity to PARPis. Mechanistically, PARP1 cooperates with the fork‐protection factors TIMELESS and TIPIN to safeguard the replisome from transcription–replication conflicts during early S phase [19]. PARP inhibition may therefore compromise the resolution of SPA‐induced replication‐associated lesions. In parallel, SPA suppresses multiple HRR genes, including an approximately fourfold reduction in *BRCA2* expression, resulting in impaired HRR without requiring genomic alterations in HRR genes [20]. Together, SPA‐induced transcriptional stress and reduced HRR capacity may create a state of functional DNA‐repair deficiency. PARP inhibition further impairs replication‐fork protection and DNA lesion resolution, leading to persistent transcription–replication conflicts, accumulation of DSBs, and ultimately cell death (Figure 1). This model offers a plausible mechanistic basis for combining SPA with PARPis, including in tumors lacking canonical HRR gene mutations. However, this hypothesis remains preliminary because mechanistic studies are limited, and substantial investigation is needed to establish how SPA and PARP inhibition interact.

**FIGURE 1:**
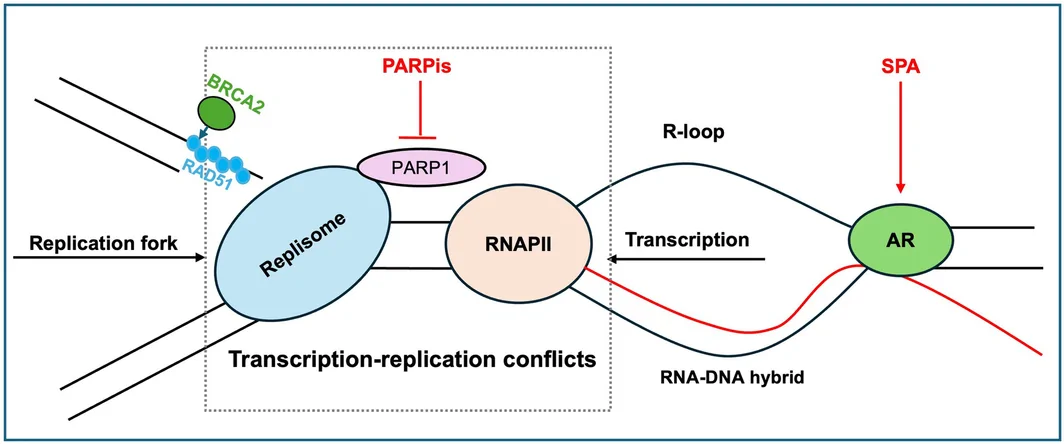
Proposed mechanistic model for SPA and PARP inhibitor combination therapy. SPA induces AR hyperactivation and excessive transcription, promoting the accumulation of R‐loop containing RNA–DNA hybrid and increasing transcription–replication conflicts. SPA‐mediated BRCA2 downregulation impairs RAD51 loading and replication‐associated homologous recombination, thereby compromising stalled‐fork protection and restart. PARP inhibition further disrupts the resolution of replication‐associated DNA lesions. Together, collisions between advancing replication forks and elongating RNA polymerase II, coupled with the accumulation of unrepaired DNA double‐strand breaks, may drive lethal genomic instability.

Clinically, this model is supported by multiple datasets. BAT deep responders (≥ 70% PSA decline; *N* = 22) are enriched for *TP53* and/or HRR alterations, with durable responses of approximately one year observed in sequenced cases [21]. A Phase II cohort of 22 patients with combined tumor suppressor loss demonstrated meaningful PSA and radiographic responses to BAT, particularly in those harboring *TP53* alterations, suggesting that tumors with impaired genome maintenance remain susceptible to androgen‐induced DNA damage [22]. A Phase II study of BAT plus olaparib in biomarker‐unselected mCRPC after ARPI therapy demonstrated PSA response rates of up to 44% and a median progression‐free survival of ~13 months, with similar efficacy in HRR‐mutated and HRR‐intact subgroups. Exploratory analyses showed only weak correlation with genomic HRD signatures, while *TP53* and HRR alterations were more consistently enriched among responders [15]. The role of p53 in AR‐mediated DNA damage responses and transcription‐coupled HRR remains incompletely defined and may differ from its canonical functions. Higher AR activity may also correlate with improved outcomes, although this requires further validation.

Although BAT has largely been studied for its ability to re‐sensitize mCRPC to subsequent antiandrogen therapy—through ARPI‐induced adaptive AR overexpression that paradoxically enhances BAT responsiveness and facilitates ARPI re‐challenge [12, 13]—the proposed BAT–PARPi synergy is AR‐dependent, suggesting that minimizing prior ARPI exposure may be important to maximize BAT‐induced DNA damage and therapeutic efficacy. Notably, BAT combined with PARPis appears to exhibit HRR‐agnostic activity through a transcription‐coupled HRD state, in contrast to conventional ARPI–PARPi combinations, which are largely restricted to *BRCA*‐mutated populations. These findings suggest that BAT‐based strategies may be better suited for earlier treatment settings in which AR dependency is preserved, thereby enhancing synthetic lethality and informing optimal therapeutic sequencing in mCRPC [23].

The combination of BAT and PARPis raises concerns regarding potential additive toxicity. SPA carries cardiovascular risks—including thromboembolic events and fluid retention—as noted in the BAT plus olaparib trial [15], while PARPis independently cause hematologic toxicities (anemia and thrombocytopenia) as well as nausea and fatigue. The combined toxicity profile has not been systematically characterized; future trials should incorporate rigorous cardiovascular and hematologic monitoring with appropriate patient selection.

Taken together, preclinical evidence suggests that BAT induces acute AR‐mediated DNA damage while simultaneously suppressing HRR gene expression, potentially converting both HRR‐deficient and HRR‐proficient tumors into a transient transcription‐coupled HRD state, thereby enhancing PARPi sensitivity. If validated prospectively, this mechanism may extend PARPi benefit beyond classical *BRCA2*‐mutant disease and mitigate resistance associated with *TP53* or *RB1* alterations, challenging the long‐standing paradigm of androgen suppression and expanding the therapeutic scope of PARPis in mCRPC. Prospective randomized trials with pre‐specified biomarker stratification and paired tumor biopsies will be essential to establish the clinical utility of BAT‐based combination strategies.

## Funding

This work was supported in part by grants from Kaohsiung Municipal Siaogang Hospital (S‐114‐04) and the Research Center for Precision Environmental Medicine at Kaohsiung Medical University, as well as by internal funding from the Department of Urology at Brigham and Women's Hospital, Boston, Massachusetts, USA.

## Conflicts of Interest

The authors declare no conflicts of interest.

## Data Availability

Data sharing not applicable to this article as no datasets were generated or analysed during the current study.
